# Evolution and development of interhemispheric connections in the vertebrate forebrain

**DOI:** 10.3389/fnhum.2014.00497

**Published:** 2014-07-14

**Authors:** Rodrigo Suárez, Ilan Gobius, Linda J. Richards

**Affiliations:** ^1^Queensland Brain Institute, The University of QueenslandBrisbane, QLD, Australia; ^2^School of Biomedical Sciences, The University of QueenslandBrisbane, QLD, Australia

**Keywords:** anterior commissure, axon guidance, commissural plate, comparative neuroanatomy, corpus callosum, hippocampal commissure

## Abstract

Axonal connections between the left and right sides of the brain are crucial for bilateral integration of lateralized sensory, motor, and associative functions. Throughout vertebrate species, forebrain commissures share a conserved developmental plan, a similar position relative to each other within the brain and similar patterns of connectivity. However, major events in the evolution of the vertebrate brain, such as the expansion of the telencephalon in tetrapods and the origin of the six-layered isocortex in mammals, resulted in the emergence and diversification of new commissural routes. These new interhemispheric connections include the pallial commissure, which appeared in the ancestors of tetrapods and connects the left and right sides of the medial pallium (hippocampus in mammals), and the corpus callosum, which is exclusive to eutherian (placental) mammals and connects both isocortical hemispheres. A comparative analysis of commissural systems in vertebrates reveals that the emergence of new commissural routes may have involved co-option of developmental mechanisms and anatomical substrates of preexistent commissural pathways. One of the embryonic regions of interest for studying these processes is the commissural plate, a portion of the early telencephalic midline that provides molecular specification and a cellular scaffold for the development of commissural axons. Further investigations into these embryonic processes in carefully selected species will provide insights not only into the mechanisms driving commissural evolution, but also regarding more general biological problems such as the role of developmental plasticity in evolutionary change.

## Introduction

In animals with bilateral symmetry, integration between the left and right sides of the body is crucial for processing lateralized sensory-motor functions. This is accomplished by axonal connections between the two sides of the nervous system, known as commissures. Commissural systems are present throughout vertebrate and invertebrate species (Arendt et al., 2008; Semmler et al., 2010), and similar mechanisms of axon guidance across the midline suggest the conservation of these developmental processes from a common bilaterian ancestor (Brose et al., 1999; Hirth and Reichert, 2007; Round and Stein, 2007; Evans and Bashaw, 2012).

During vertebrate evolution, several brain developmental events have been conserved from lampreys to humans, possibly explaining the broad anatomical similarity of adult forebrain commissures across species. However, diversification of the telencephalic commissures in mammals, including new axonal routes in diprotodont marsupials and the origin of the corpus callosum in eutherian (placental) mammals, illustrate natural examples of diversity in the developmental mechanisms involved in commissure formation.

Development of commissures entails a sequence of events involving morphogenic area patterning, cell-type specification, neuron-glia interactions, production and reception of guidance cues, axonal growth and navigation, and activity-dependent establishment of contralateral connections. In humans, disorders affecting these events at any stage can prevent the normal formation of the commissures, resulting in mild to severe sensory-motor and cognitive conditions (for specific review, see Paul et al., 2007). Therefore, understanding the fundamental processes directing commissure formation remains an important challenge for neuroscientists. One way to address this includes adopting an evolutionary-developmental perspective, i.e., to compare experimental data on commissure development and function from different species while considering the phylogenetic relationships between them. This allows the categorization of developmental processes as conserved or derived within lineages, thus outlining critical features of normal brain development. Using this approach, here we examine anatomical and developmental features of forebrain commissures in vertebrates to gain insights into the development and evolution of the corpus callosum, the largest axonal tract in the human brain.

## Conservation of a developmental plan in the vertebrate brain

**Figure 1:**
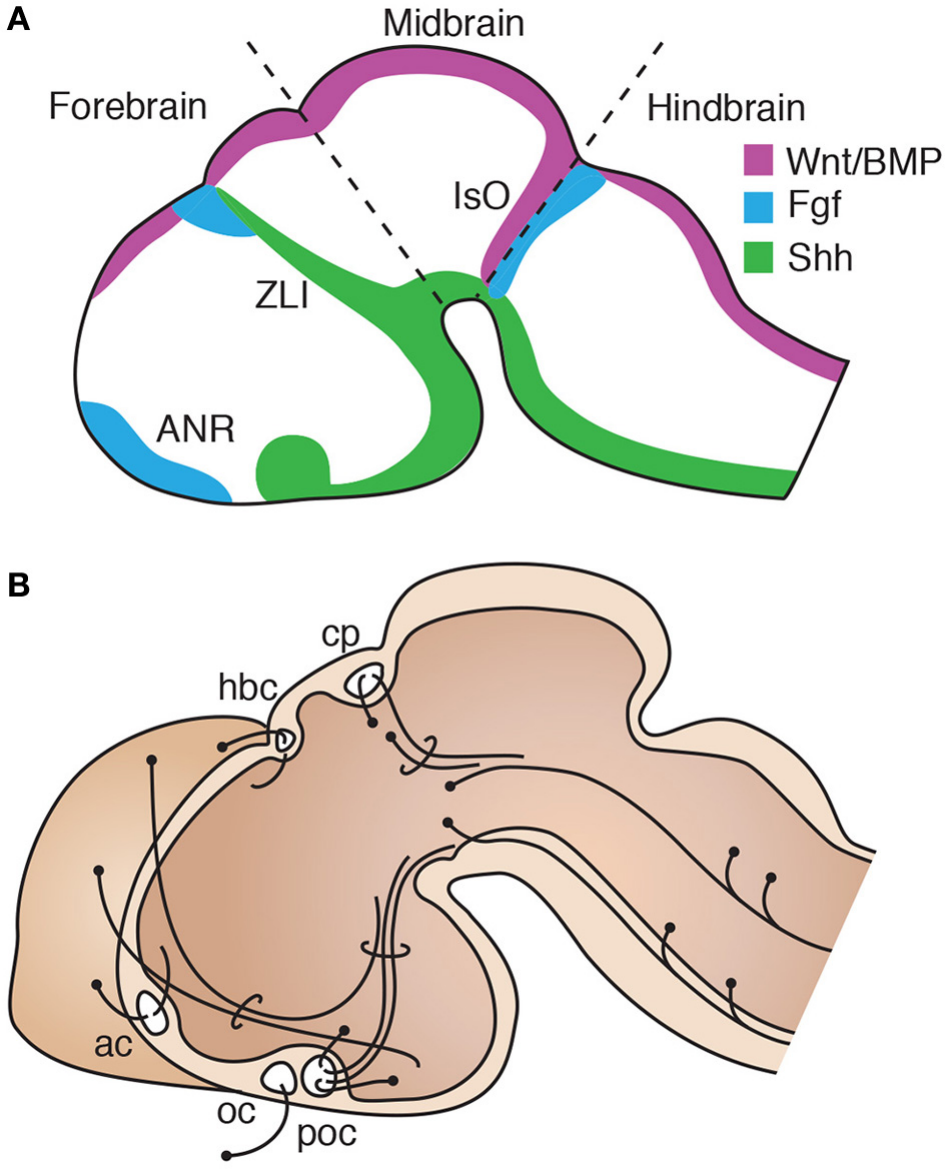
**Conservation of a general organization of vertebrate brain development. (A)** Diagram of an early stage of brain development in a model vertebrate, equivalent to mouse E11, showing the principal regions of morphogen expression. Rostral expression of Fgf defines the anterior neural ridge (ANR). The *zona limitans intrathalamica* (ZLI) is defined by a narrow band of Shh expression, with Fgf and BMP/Wnt coexpression dorsally at the border between the presumptive telencephalon and diencephalon. Caudally, the isthmic organizer (IsO) marks the boundary between the midbrain and hindbrain territories. **(B)** Midsagittal schematic of a model vertebrate brain at a later stage, equivalent to mouse E14, showing the position of the first axon bundles that form during development, including the posterior commissure (cp) and post-optic commissure (poc), followed by the anterior commissure (ac), habenular commissure (hbc) and optic chiasm (oc). Dorsal is to the top and rostral to the left.

The origin and diversification of forebrain commissures in vertebrates is likely to be related to a general developmental plan upon which evolution may act. Such is the case of the early molecular determination of midline forebrain territories, which is strikingly similar across vertebrate species. It involves the patterned expression of morphogens in defined regions that, through their interaction in three-dimensional space, specify cellular fate and commissure formation. After closure of the neural tube, patterning centers at the dorsal and ventral midline establish gradient territories through the expression of the diffusible morphogens Wnt/BMP and sonic hedgehog (Shh), respectively. At the rostral tip of the prosencephalon, fibroblast growth factor (Fgf) proteins are expressed in a region known as the anterior neural ridge, which then becomes the commissural plate, a structure through which the telencephalic commissures cross the midline (Figure 1A). Fgfs are also expressed more caudally along the dorsal midline, at the border between the presumptive prethalamus and dorsal thalamus, in a patterning region known as the *zona limitans intrathalamica*, which is characterized by a narrow band of Shh expression that forms a continuum with ventral Shh expression in the prechordal plate. The isthmic organizer, another patterning center widely conserved in vertebrates, is located at the border between the midbrain and hindbrain and is characterized by a narrow ring of Fgf and Wnt/Bmp expression extending dorsoventrally (Figure 1A). This general organization is largely maintained across vertebrate taxa from lampreys to mammals (Walshe and Mason, 2003; Buckles et al., 2004; Wilson and Houart, 2004; Tole et al., 2006; O'Leary et al., 2007; Rétaux and Kano, 2010; Rash and Grove, 2011; Sugahara et al., 2013), and therefore represents an important landmark in brain development. Moreover, the relative positions and expression profiles of these patterning centers are similarly present in some non-vertebrate lineages, such as the hemichordate acorn worm, suggesting the ancient conservation of a morphogenic program since early deuterostomes (Pani et al., 2012). Notably, these early systems of protein gradient production not only instruct overall brain area patterning (Shimogori and Grove, 2005; O'Leary et al., 2007; Assimacopoulos et al., 2012), but also serve as guidance cues for growing axons (Charron et al., 2003; Walshe and Mason, 2003; Tole et al., 2006; Zou and Lyuksyutova, 2007; Toyama et al., 2013). Similarly, as described in more detail below, the spatial locations of these organizing centers broadly coincide with regions of commissural axon crossing, such as the post-optic commissure and posterior commissure, which are the first commissures to form during vertebrate development (Figure 1B; Herrick, 1937; Kuratani et al., 1998; Doldan et al., 2000; Barreiro-Iglesias et al., 2008). Thus, the conservation of these early mechanisms of forebrain development across vertebrate species suggest that area patterning and cell-specification functions may have been co-opted for axon guidance and commissural circuit formation. Therefore, the emergence of non-disruptive variations in these processes may underlie the evolution of commissural diversity.

## Conserved commissural pathways in early vertebrates

**Figure 2:**
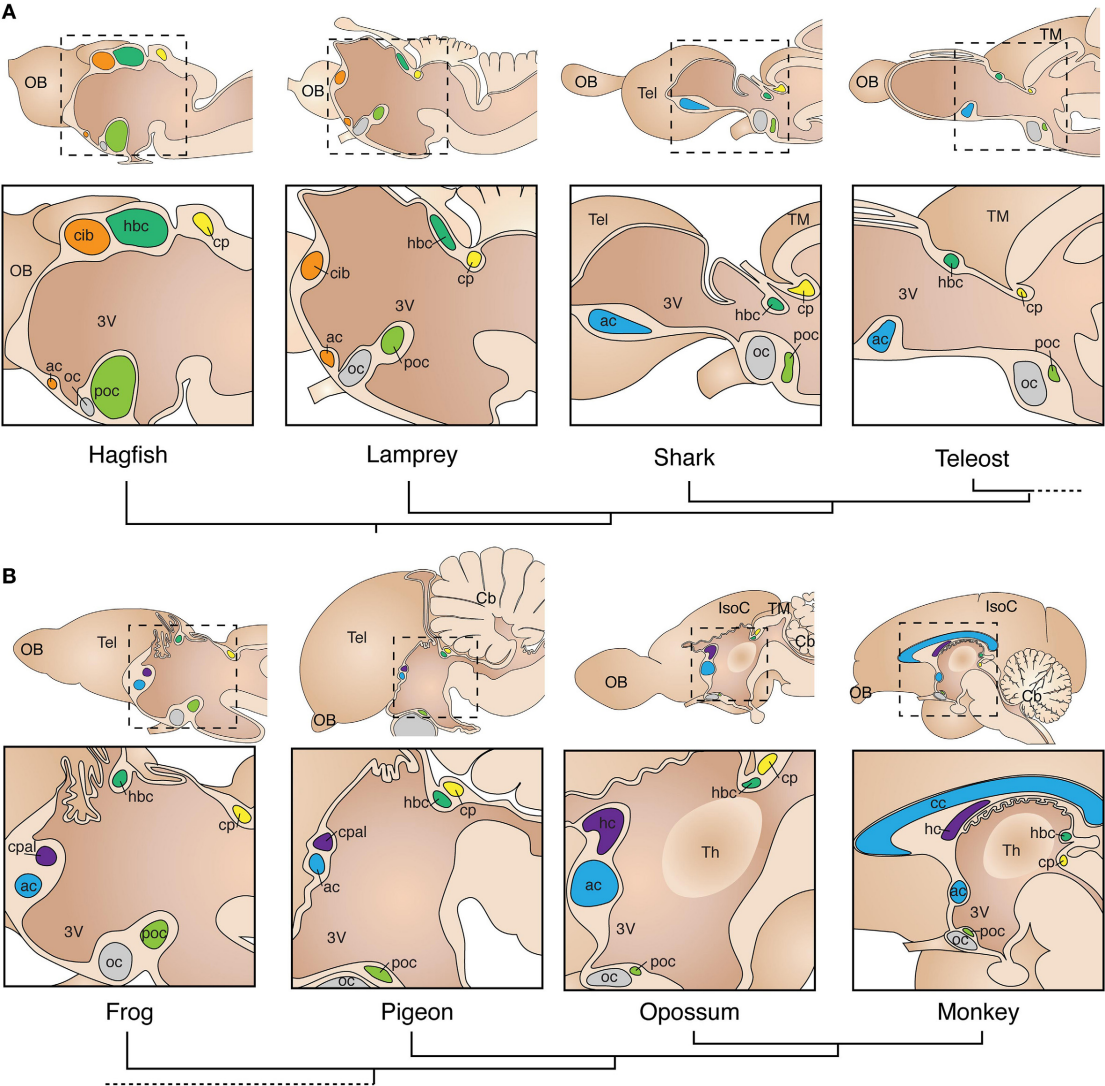
**Conservation of commissural systems across adult vertebrate species. (A)** Commissures in non-tetrapod species. Note the conserved position of commissures relative to each other within and between species, commissures are color-coded according to homology hypotheses. The *commissura interbulbaris* (cib) and anterior commissure (ac) of lampreys and hagfish are depicted here with a unique color (orange) to indicate the uncertainty of definitive homology with other vertebrates. **(B)** Tetrapods are characterized by the evolution of a distinct pallial commissure (cpal) in close dorsal proximity with the anterior commissure. The mammalian homolog of the pallial commissure is known as hippocampal commissure (hc). The corpus callosum (cc) is an evolutionary innovation of placental mammals, located dorsal to the hippocampal commissure. Phylogenetic relationships between species are depicted with dendrograms below species name. 3V, third ventricle; Cb, cerebellum; cp, posterior commissure; hbc, habenular commissure; IsoC, isocortex; OB, olfactory bulb; oc, optic chiasm; poc, post-optic commissure; Tel, telencephalon; Th, thalamus; TM, tectum mesencephali.

To examine commissural diversity and evolution, we will first refer to the anatomical organization of forebrain commissures in early-branched vertebrates. A gross comparison of the brain of the jawless hagfish and lampreys, cartilaginous sharks, and teleost fish, reveals overall similarities in the relative position of commissural connections within the brain (Figure 2A). Briefly, at the caudal-most extent of the forebrain lies the posterior commissure (cp; Figure 2A, yellow), which connects dorsal regions of the diencephalon (i.e., dorsal thalamus) and mesencephalon (i.e., pretectum and optic tectum) (Nieuwenhuys and Nicholson, 1998; Wicht and Nieuwenhuys, 1998). In the basal diencephalon, two regions of midline axon crossing are found throughout vertebrates: the postoptic commissure (poc; Figure 2, light green), and optic chiasm (oc; Figure 2, gray). The postoptic commissure carries axons bilaterally connecting the preoptic area and the hypothalamus, as well as telencephalic and thalamic fibers projecting to the hypothalamic region (Nieuwenhuys and Nicholson, 1998; Smeets, 1998; Wicht and Nieuwenhuys, 1998). In all vertebrates, axons from retinal ganglion cells decussate, at least partially, at the optic chiasm to terminate in contralateral diencephalic (lateral thalamus, hypothalamus) and mesencephalic (pretectum, tectum) targets. However, as axons forming the optic tract decussate *en route* to their central targets, without reciprocally connecting bilateral regions, the optic chiasm is not considered a proper commissure. Along the roof of the midline, immediately rostral to the posterior commissure, lies the habenular commissure (hbc; Figure 2A, green), which is prominent in agnathans as compared to other vertebrates (Wicht and Northcutt, 1992). The habenular commissure connects the epithalamus bilaterally, and also contains axons originating from the olfactory bulbs and medial pallium (*olfacto-habenularis* tract) that terminate contralaterally in pallial, subpallial and diencephalic targets (Northcutt and Puzdrowski, 1988; Polenova and Vesselkin, 1993). The largest commissure in the telencephalon of agnathans is the *commissura interbulbaris* (cib, Figure 2A, orange). It carries fibers from the olfactory bulbs and pallium, thus resembling the rostral component of the habenular commissure. In fact, the *commissura interbulbaris* and habenular commissure are located in close proximity to each other in hagfish, and it is hard to distinguish fibers crossing through one or the other commissure (Wicht and Northcutt, 1992, 1998; Wicht and Nieuwenhuys, 1998). In contrast, lampreys have a relatively smaller *commissura interbulbaris*, located more rostral to the habenular commissure than hagfishes (Figure 1A; Northcutt and Puzdrowski, 1988; Polenova and Vesselkin, 1993; Nieuwenhuys and Nicholson, 1998; Pombal et al., 2009). This difference may relate to the fact that while hagfish undergo direct development with olfactory-guided swimming occurring throughout ontogeny, lampreys spend several years as a sessile larva buried in mud, with olfactory behaviors becoming active only during their brief adulthood. Thus, the seemingly derived behavioral and neuroanatomical features of extant agnathans makes it difficult to formulate hypotheses regarding homology of their telencephalic commissural circuits with those of other vertebrates (see Table 1).

**Table 1:** **Comparison of interhemispheric connections through telencephalic commissures in vertebrates**.

	**Agnatha (Jawless vertebrates; e.g., hagfish and lampreys)**	**Gnathostomata (Jawed vertebrates)**						
		**Chondrichthyes (cartilaginous fish; e.g., sharks and rays)**	**Teleosts (ray-finned fish; zebrafish and goldfish)**	**Sarcopterygii (lobe-finned vertebrates)**				
				**Lungfish**	**Reptiles**	**Birds**	**Mammals**	
							**Marsupials**	**Eutherians**
Anterior commissure	Olfactory recipient nuclei and septum to contralateral homotopic regions and hypothalamus^[1, 2]^.	Olfactory bulbs to contralateral retrobulbar area, septum and striatum^[5, 6]^.	Olfactory bulbs, pallial and subpallial areas to contralateral homotopic regions^[7, 8]^.	Olfactory recipient and subpallial septum to contralateral homotopic regions^[9]^.	Olfactory recipient and basal telencephalon to homotopic regions^[10, 11]^.	Olfactory recipient and basal telencephalon to homotopic regions^[14]^.	Olfactory recipient, basal telencephalic, piriform cortex and isocortex to homotopic regions^[16]^.	Olfactory recipient, basal telencephalic, piriform cortex and temporal isocortex to homotopic regions^[18, 19]^.
*Commissura interbulbaris*/pallial commissure/Hippocampal commissure	(*Commissura interbulbaris*) Olfactory bulbs and pallium to contralateral homotopic, subpallial and diencephalic targets^[1, 3, 4]^.	–/?	–/?	(Pallial commissure) Medial pallium to contralateral medial and dorsal pallium^[9]^.	(Pallial commissure) Medial and dorsal pallium to contralateral homotopic regions^[11, 12, 13]^.	(Pallial commissure) Medial pallium to contralateral homotopic regions^[15]^.	(Hippocampal commissure) Hippocampus to contralateral homotopic regions^[17]^.	(Hippocampal commissure) Hippocampus to contralateral homotopic regions and entorhinal cortex^[20, 21]^.
Corpus callosum	–	–	–	–	–	–	–	Cingulate cortex and most of isocortex to contralateral homotopic regions^[21]^.

References:

1 Nieuwenhuys and Nicholson, 1998;

2 Wicht and Nieuwenhuys, 1998;

3 Northcutt and Puzdrowski, 1988;

4 Polenova and Vesselkin, 1993;

5 Smeets, 1983;

6 Yáñez et al., 2011;

7 Folgueira et al., 2004;

8 Northcutt, 2006;

9 Northcutt and Westhoff, 2011;

10 Lanuza and Halpern, 1997;

11 Butler, 1976;

12 Voneida and Ebbesson, 1969;

13 Martínez-García et al., 1990;

14 Zeier and Karten, 1973;

15 Atoji et al., 2002;

16 Ashwell et al., 1996a;

17 Smith, 1937;

18 Ramón y Cajal, 1904;

19 Van Alphen, 1969;

20 Wyss et al., 1980;

21 *Yorke and Caviness, 1975*.

At the rostral-most extent of the midline lies the anterior commissure, which in agnathans connect mostly the olfactory bulbs and septum with their contralateral homotopic structures, as well as with hypothalamic targets (Nieuwenhuys and Nicholson, 1998; Wicht and Nieuwenhuys, 1998). Similarly, in cartilaginous fish such as sharks and rays, the anterior commissure carries axons connecting the olfactory bulbs bilaterally, as well as with the septum and striatum (Smeets, 1983, 1998; Yáñez et al., 2011). Interestingly, secondary olfactory axons of cartilaginous and bony fish decussate not only through the anterior commissure, but also through the habenular and postoptic commissures (Smeets, 1998; Northcutt, 2011; Yáñez et al., 2011), suggesting that decussating axons from a single region may cross the midline using more than one commissural route. Whether the medial pallium of sharks and rays connects to contralateral homotopic regions through any of these commissures is not fully established. However, a general pattern of telencephalic connections through the anterior commissure linking olfactory, pallial and subpallial structures is also observed in ray-finned bony fish (Table 1; Folgueira et al., 2004; Northcutt, 2006, 2011). Ray-finned fish are characterized by a developmental eversion of the telencephalon, which contrasts with the evagination of the telencephalic vesicles observed in all other vertebrates, where the homologs of the medial pallium develop into the lateral-most part of the telencephalon (for specific reviews, see Meek and Nieuwenhuys, 1998; Northcutt, 2008; Nieuwenhuys, 2009). This telencephalic arrangement may have prevented the evolution of a defined pallial commissure (which connects the medial pallium in tetrapods, see below) at the dorsal midline in this group. However, in goldfish, axons arising from the homolog of the medial pallium (ventro-lateral portion of the *area dorsalis*), cross the midline at more dorsal territories within the anterior commissure than axons from the olfactory pallium (medial portion of the *area dorsalis*), which decussate more ventrally within the anterior commissure (Northcutt, 2006). Notably, this dorso-ventral parcellation of fibers according to the location of their cell bodies is a feature also present in the telencephalic commissures of tetrapods (see next section). Thus, a topographical arrangement of commissural fibers seems to predate the segregation and emergence of new discrete commissures. In summary, a basic configuration of commissural systems has been conserved since early vertebrates, including the coexistence of homotopic and heterotopic connections within commissural tracts, as well as a spatially segregated arrangement of axons according to their site of origin. Both anatomical features are further evident in the telencephalic commissures of tetrapods.

## Origin and diversification of pallial commissures

**Figure 3:**
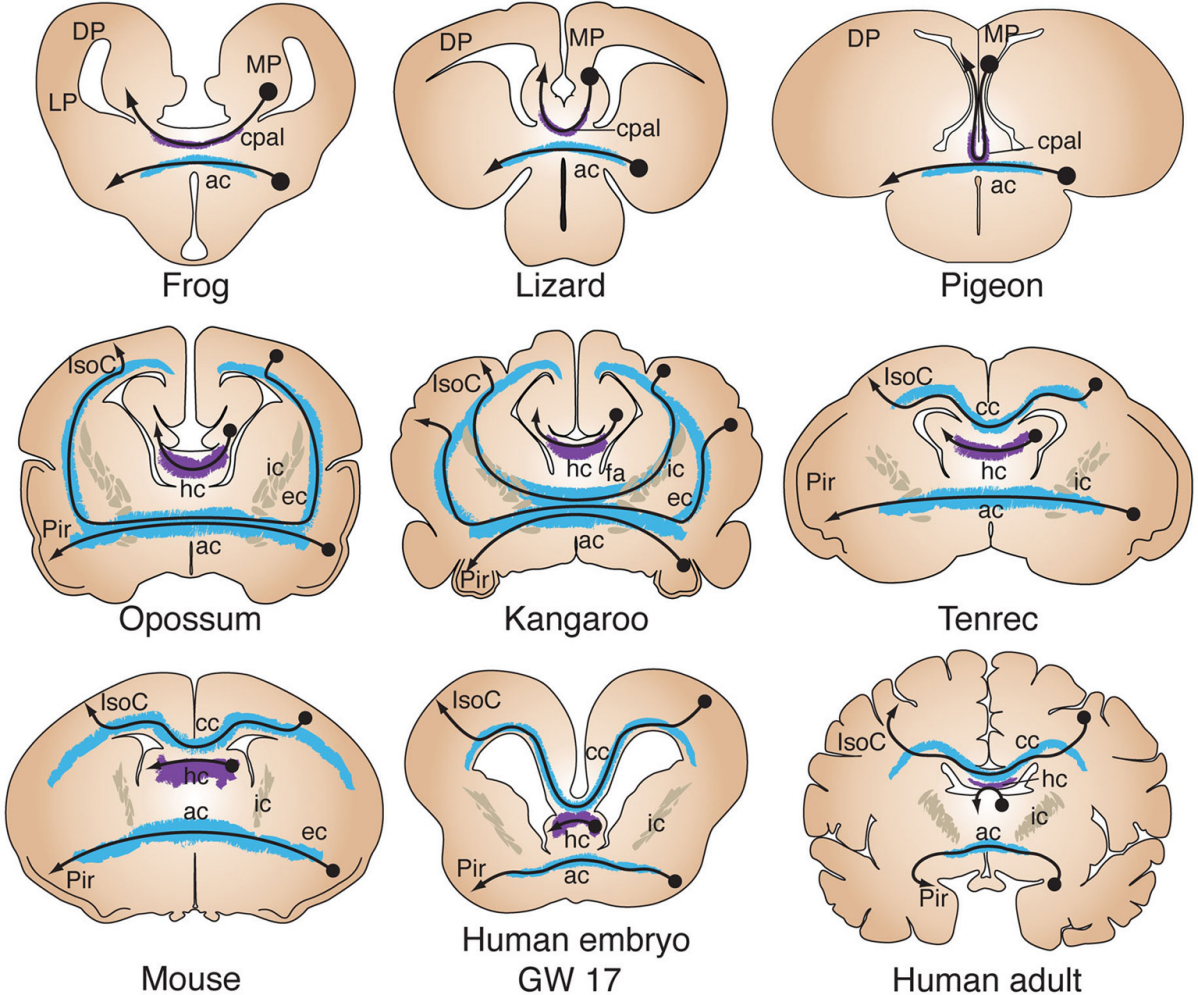
**Evolution of telencephalic commissures in tetrapods**. Coronal schematics of tetrapod brains show the close association between the pallial commissure (cpal) and the anterior commissure (ac), bilaterally connecting the medial pallium (MP) and olfactory recipient structures, respectively. In the opossum all isocortical (IsoC) and piriform (Pir) commissural projections cross through the anterior commissure (ac) after coursing through the external capsule (ec). In the kangaroo, as in other diprotodont marsupials, axons from more dorsal regions of the isocortex course through the internal capsule (ic) toward the anterior commissure, forming the *fasciculum aberrans* (fa). Hippocampal neurons decussate through the hippocampal commissure (hc). In tenrecs, as in other basal placentals with a small IsoC/Pir ratio, the corpus callosum (cc) is a small structure located immediately above the hippocampal commissure. Developmental studies in mice and humans have shown that all three commissures arise from the commissural plate, forming a single plane of morphogenic patterning. GW, gestational week; DP, dorsal pallium; LP, lateral pallium.

A crucial milestone in vertebrate evolution that resulted in several behavioral and anatomical adaptations, including a significant increase in brain complexity, was the colonization of terrestrial niches by the ancestors of modern tetrapods. In particular, the telencephalic pallium underwent considerable increase in size and number of connections, acquiring further complexity in mammals with the evolution of the six-layered isocortex. Consequently, the telencephalon of tetrapods evolved additional commissures that provide interhemispheric connections between pallial regions. Early neuroanatomists described a distinct commissure in the telencephalon of reptiles, termed the pallial commissure (cpal; Figure 2B, purple; Herrick, 1910; Johnston, 1913). This structure connects mainly the left and right portions of the medial pallium, which in mammals gives rise to the hippocampal formation (Table 1; Voneida and Ebbesson, 1969; Butler, 1976; Kokoros and Northcutt, 1977; Martínez-García et al., 1990; Atoji et al., 2002; Northcutt and Westhoff, 2011). The oldest indication of a distinct pallial commissure in vertebrates comes from the spotted African lungfish, a basal member of the lineage of lobe-finned fish that includes all tetrapods and their common ancestor (Sarcopterygii). In lungfish, the pallial commissure is located immediately rostro-dorsal to the anterior commissure. It differs from the anterior commissure by its medial pallial, as compared to subpallial, bilateral connections (Northcutt and Westhoff, 2011). Similarly, the telencephalic commissures of amphibians include bilateral connections from subpallial and olfactory-recipient nuclei through the anterior commissure, and medial pallial connections through the dorsally-located pallial commissure (Figure 3; Kokoros and Northcutt, 1977; Hofmann and Meyer, 1989; Northcutt and Ronan, 1992). This fiber topography in lungfish and amphibians, along with the axonal parcellation of the anterior commissure of teleost fish, suggest that the evolution of the pallial commissure likely involved a transition from dorsally-fasciculated medial pallial axons within the anterior commissure, to a more defined dorsal segregation of fibers within the rostral tip of the *lamina terminalis* (see Figures 2B, 3). Accordingly, both commissures arise from the same embryonic territory, the commissural plate (see next section).

Sensory adaptations may also have influenced the evolution and diversification of telencephalic connections, including commissural systems. Colonization of land involved the evolution of aerial respiration and the emergence of an accessory olfactory system specialized in pheromone detection (for a review, see Suárez et al., 2012). In non-mammalian sarcopterygians, efferents from the main and accessory olfactory bulbs decussate through different commissural routes, i.e., the habenular and anterior commissure, respectively (Halpern, 1976; Ulinski and Peterson, 1981; Martinez-Garcia et al., 1991; Scalia et al., 1991; Lohman and Smeets, 1993; Lanuza and Halpern, 1997; Moreno et al., 2005; Patzke et al., 2011; Northcutt and Rink, 2012; Atoji and Wild, 2014), suggesting that the diversification of decussated sensory input to the telencephalon may have also affected the rearrangement of commissural systems.

Similar connectivity patterns are found in amniotes, such as reptiles and birds, where the anterior commissure connects mostly subpallial and olfactory-recipient regions from both hemispheres (Zeier and Karten, 1973; Butler, 1976; Lanuza and Halpern, 1997), whereas the pallial commissure carries axons connecting mostly the dorsal septum and topographically arranged fibers of the hippocampus (Table 1; Voneida and Ebbesson, 1969; Butler, 1976; Martínez-García et al., 1990; Atoji et al., 2002). Accordingly, since its discovery the pallial commissure has been considered homologous to the hippocampal commissure of mammals (Figures 2B, 3; Herrick, 1910; Johnston, 1913). In mammals, the pallial commissure has received the names of hippocampal commissure, psalterium and crus (or decussation) of the fornix. It connects mostly homotopic regions of the hippocampus *cornu ammonis* between hemispheres, as well as heterotopic fibers connecting the hippocampus with the entorhinal cortex (Steward, 1976; Wyss et al., 1980; Voneida et al., 1981; Cui et al., 2013). The evolution of the six-layered isocortex in mammals correlates with a further increase in size and complexity of telencephalic commissures. For example, the corpus callosum, the largest axon tract in the human brain, is a relatively recent evolutionary innovation exclusive to placental mammals. Richard Owen, a prominent anatomist contemporary to Darwin, provided the first comparative study of telencephalic commissures in mammals. He discovered that marsupials lack a corpus callosum, and that their telencephalic commissures include exclusively the hippocampal and anterior commissures, referring to the commissural system of marsupials as “… *a structure of brain which is intermediate of that between placental Mammalia and Birds*” (Owen, 1837; p. 92). In monotremes and non-diprotodont marsupials all interhemispheric isocortical connections reach the anterior commissure via the external capsule, whereas diprotodont marsupials, such as koalas and kangaroos, possess an additional axonal tract, termed the *fasciculus aberrans*, that joins the dorsal aspect of the anterior commissure through the internal capsule (Figure 3; Flower, 1865; Smith, 1897, 1902, 1937; Johnston, 1913; Abbie, 1939; Ashwell et al., 1996a). Again, this topographic arrangement of commissural fibers may reflect a common feature of commissural systems. Interestingly, the evolution of the corpus callosum as the main pathway for isocortical and cingulate commissural connections in eutherians resulted in the anterior commissure reverting to its ancestral state, i.e., connecting mostly olfactory recipient and subpallial nuclei. Still, some axons from lateral portions of the temporal isocortex decussate via the anterior commissure (Ramón y Cajal, 1904; Horel and Stelzner, 1981; Jouandet and Hartenstein, 1983; Tomasi et al., 2012).

**Figure 4:**
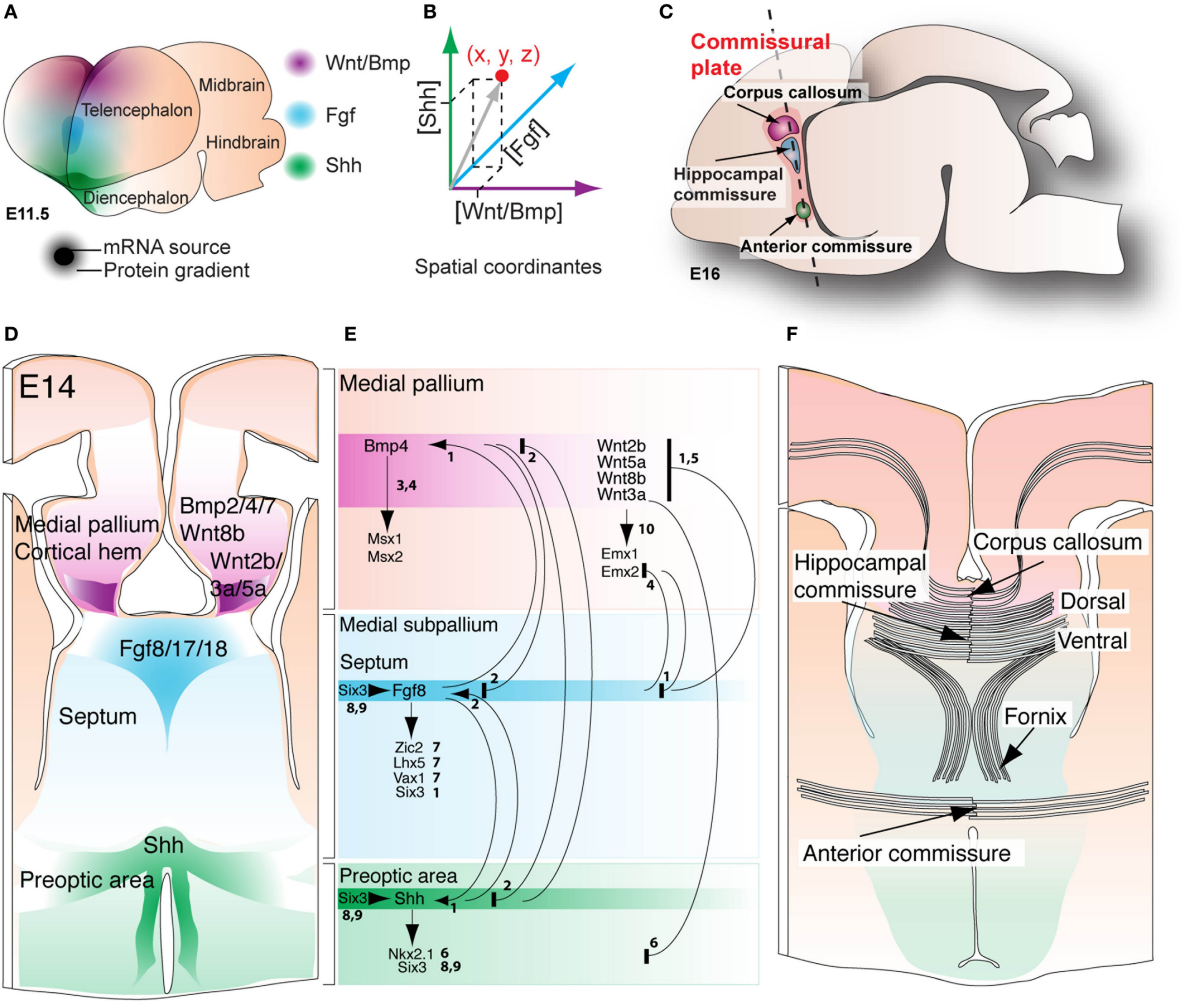
**Morphogenic patterning at the commissural plate. (A)** Discrete regions of the early telencephalic midline of mice at E 11.5 express diffusible Wnt/Bmp, Fgf, and Shh proteins, as revealed by mRNA expression studies. **(B)** The differential concentration of each morphogen at any point in space results in distinct intracellular signaling outcomes, generating different cell fates. **(C)** A midsagittal schematic of the embryonic mouse brain showing the plane of section **(D,F)** defined by telencephalic commissures, known as the commissural plate. **(D)** Transverse section through the presumptive commissural plate at E14 shows the spatial extent of morphogen expression, mostly defining pallial, septal, and preoptic domains. **(E)** In general, morphogen interactions are reciprocally repressive between the pallial and subpallial regions; numbers denote references providing evidence for each interaction (see below for reference key). Further definition of the medial pallium, septum and preoptic areas is achieved by the induction of transcription factors such as Msx1/2, Emx1/2 (pallial), Zic2, Lhx5, Vax1 (septal), Six3 and Nkx2.1 (preoptic). **(F)** Dorso-ventral patterning domains also define the dorso-ventral level at which the three telencephalic commissures will cross within the caudal telencephalic midline. References: ^1^Storm et al., 2006; ^2^Ohkubo et al., 2002; ^3^Fernandes et al., 2007; ^4^Hebert et al., 2003; ^5^Shimogori et al., 2004; ^6^Gunhaga et al., 2003; ^7^Okada et al., 2008; ^8^Geng et al., 2008; ^9^Jeong et al., 2008; ^10^Lee et al., 2000.

The events that led to the evolution of the mammalian isocortex in general, and eutherian corpus callosum in particular, cannot be fully understood from the fossil record and therefore require comparative developmental and molecular approaches. However, fossil skull endocasts of early ancestors of modern mammals suggest that the primitive mammalian brain was dominated by olfactory structures, including a large piriform cortex, and a small isocortex (Rowe et al., 2011). In modern placental mammals with a small isocortex/piriform cortex ratio, such as hedgehogs (Eulipotyphla), bats (Chiroptera) or tenrecs (Afrosoricida), the corpus callosum is very a small structure located just above the hippocampal commissure (Figure 3), possibly resembling a primitive state of early eutherians (Flower, 1865; Smith, 1897; Abbie, 1939; Krubitzer et al., 1997). Consequently, a larger corpus callosum is found in species with a higher isocortex/piriform cortex ratio, such as rodents and primates (Figures 2B, 3), suggesting that isocortical expansion explains the increase of corpus callosum size. The developmental time course of midline crossing of commissural axons in different species may also shed light on the evolution of commissures. For example, in wallabies, the anterior commissure forms first, followed by the *fasciculus aberrans* and finally the hippocampal commissure, whereas in placental mammals the anterior commissure forms first, followed by the hippocampal commissure and then the corpus callosum (Ashwell et al., 1996b). These developmental sequences suggest that the evolution of the corpus callosum involved a rerouting of dorsal cortical axons, from crossing through the anterior commissure to employing the same embryonic substrate as the hippocampal commissure. Although the developmental events that led to the evolution of the corpus callosum in placental mammals remain largely unknown, the formation of all three commissures in these species depends on the development of the commissural plate (Smith, 1897; Rakic and Yakovlev, 1968; Moldrich et al., 2010). This embryonic structure has been studied in mice and humans (Figures 3, 4), and the molecular and cellular events that characterize its development are discussed below.

## Molecular specification of the commissural plate

As discussed previously, patterning of the telencephalic midline in mouse embryos, including the establishment of dorso-ventral territories of commissure formation, is directed by the spatially defined expression of a conserved set of morphogens. The medial pallium/cortical hem expresses Wnt/BMPs, the basal prechordal plate expresses Shh, and the anterior neural ridge, or presumptive commissural plate, expresses Fgfs (Figure 4A; Rubenstein et al., 1998; Campbell, 2003; Hebert and Fishell, 2008; Borello and Pierani, 2010). These morphogens interact via gradients of protein expression, whereby the relative concentration of each morphogen differs at each point of the extracellular space, resulting in either activation or suppression of intracellular effector pathways (Figures 4A,B). In particular, the precise patterning of dorso-ventral domains at the telencephalic midline is critical for the formation of all three telencephalic commissures. Formation of the commissural plate involves the thickening of the *lamina terminalis*, whereby providing a substrate for convergence and decussation of commissural axons (Figures 4C–F; Rakic and Yakovlev, 1968; Moldrich et al., 2010). From dorsal to ventral, the earliest subdivisions of the commissural plate include the cortical hem/medial pallium, the septum, and the preoptic area, where Wnt/Bmp, Fgf and Shh signaling, respectively, induce formation of these tissues in a concentration-dependent manner (Figure 4D; see for review Rubenstein et al., 1998; Campbell, 2003; Puelles and Rubenstein, 2003; Hebert, 2005; Fernandes and Hebert, 2008; Hebert and Fishell, 2008). The formation of borders within this primordial tissue is primarily controlled by either repressive or inductive mechanisms between individual morphogen signals. For example, studies in mice and chickens have described reciprocal repression between the Bmp/Wnt and Fgf signaling pathways, and between the Bmp/Wnt and Shh signaling pathways (Figure 4E; Lee et al., 2000; Ohkubo et al., 2002; Shimogori et al., 2004; Storm et al., 2006). In contrast, Fgf8 and Shh regulate the expression of one another to maintain normal expression levels, suggesting that a reciprocal inductive mechanism is in place between the septum and preoptic areas (Ohkubo et al., 2002; Storm et al., 2006). This reciprocity between Fgf8 and Shh signaling may be integrated by the transcription factor Six3, as it can directly bind and activate a forebrain-specific *Shh* enhancer, and can also regulate the expression of Fgf8 prior to telencephalic midline formation (Lagutin et al., 2003; Geng et al., 2008; Jeong et al., 2008). Moreover, following initial telencephalic midline formation, expression of Shh and Fgf8 in the subpallium maintains Six3 expression in the septum and preoptic area (Figure 4E; Storm et al., 2006; Geng et al., 2008). Once morphogenic patterning of the commissural plate has been established, tissue-specific transcription factors further affect cell fate identity, demarcating all three dorso-ventral domains (Figure 4E). First, the medial pallium is defined by expression of transcription factors such as Emx1 and Emx2 (regulated by Wnt signaling), as well as Msx1 and Msx2 (regulated by BMP signaling) (Lee et al., 2000; Hebert et al., 2002, 2003; Shimogori et al., 2004; Fernandes et al., 2007; Caronia et al., 2010). The subpallial septum is defined by the transcription factors Zic2, Vax1, and Lhx5, where ectopic Fgf8 signaling is sufficient to induce their expression, even in the absence of Shh (Okada et al., 2008). Finally, the preoptic area expresses Six3 and Nkx2.1 under control of Shh signaling, which is essential for the formation of the entire subpallium (Figure 4E; Patten and Placzek, 2000; Ohkubo et al., 2002; Corbin et al., 2003; Gunhaga et al., 2003; Nery et al., 2003; Xu et al., 2005, 2008; Gulacsi and Anderson, 2006; Fogarty et al., 2007; Butt et al., 2008; Garcia-Lopez et al., 2008; Geng et al., 2008; Lavado et al., 2008; Gelman et al., 2009; Hirata et al., 2009; Flandin et al., 2011). Finally, another transcription factor, Gli3, has also been shown to regulate cell-type patterning within the commissural plate (Magnani et al., 2012; Amaniti et al., 2013). Loss of Gli3 affects the expression of BMP/Wnt and Fgf8 at the midline, as well as the expression of their downstream effectors, including Emx1 and Emx2 (Theil et al., 1999; Kuschel et al., 2003; Magnani et al., 2012). Although Gli3 is a known downstream effector of Shh signaling, its precise role in the integration of multiple morphogenic signals remains unclear.

Collectively, these genetic patterning studies suggest that initial formation of the commissural plate involves the morphogenic activity of BMP/Wnt and Shh to establish pallial and subpallial territories, respectively, and that the subpallium is then further refined into septal and preoptic regions through Fgf8 signaling. Thus, the specific location through which commissural axons cross the midline depends on the early molecular patterning of the commissural plate, whereby pioneer axons of the corpus callosum cross through the same pallial domain of the dorsal hippocampal commissure, while the ventral hippocampal and anterior commissures form at the septal and preoptic domains, respectively (Figure 4F; Moldrich et al., 2010). Taken together, comparative and molecular data suggest that evolution of the corpus callosum involved a rerouting of commissural axons through a preexistent pallial commissural course.

## Commissural axon guidance and contralateral targeting

**Figure 5:**
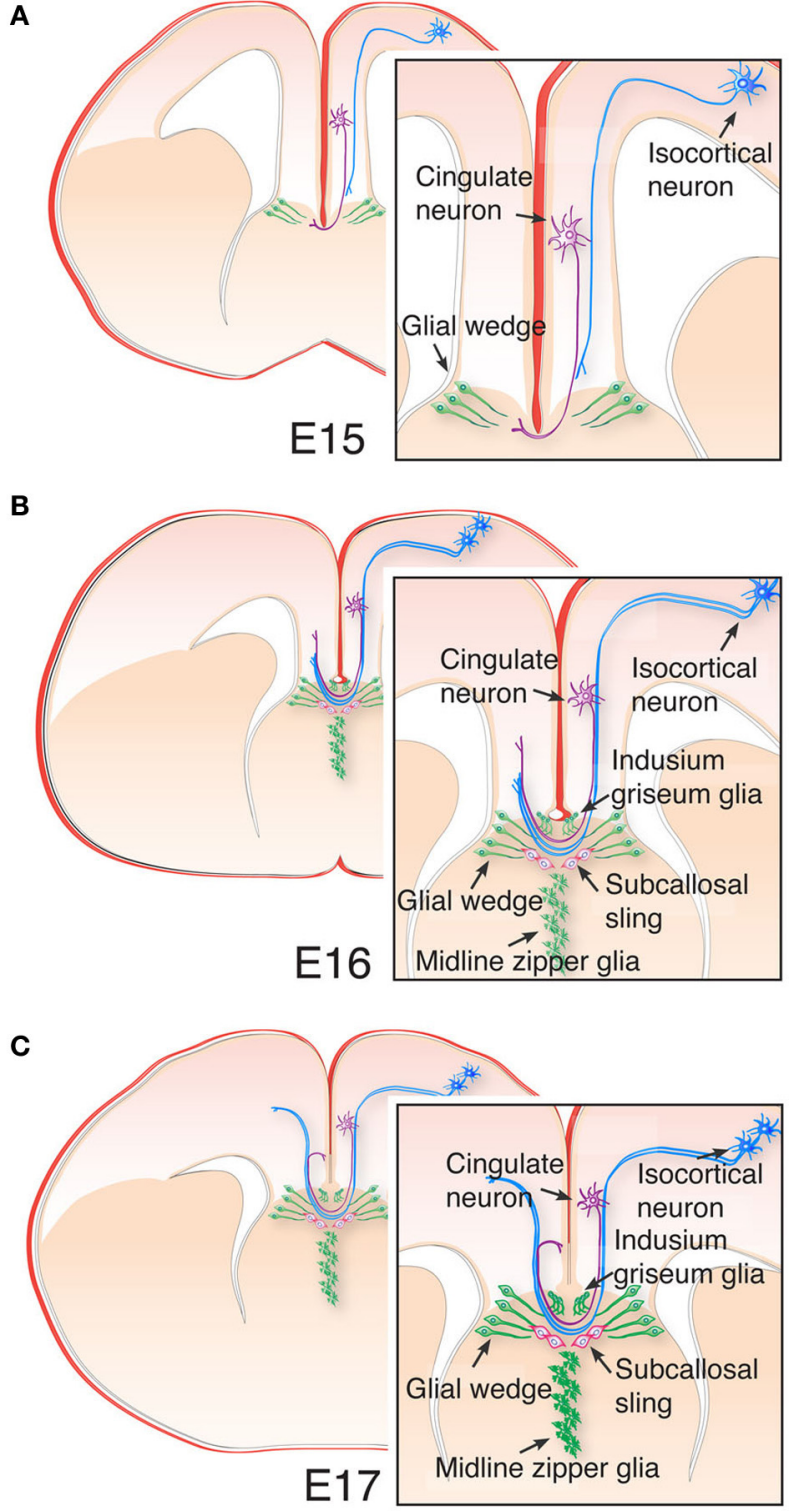
**Cellular architecture of the telencephalic midline and callosal development. (A)** The ventral-most boundary of the corpus callosum is established by glial wedge cells, as cingulate pioneering axons first cross the midline at E15, while the more laterally located isocortical axons grow toward the midline following cingulate axons. **(B)** At E16, a small number of isocortical axons have crossed the midline, and the indusium griseum glia and midline zipper glia are now detectable with Gfap immunohistochemistry. The indusium griseum glia provide the dorsal boundary of the corpus callosum. In addition, cells of the subcallosal sling begin to migrate toward the midline, just beneath the corpus callosum. **(C)** By E17, isocortical axons have started crossing the midline, and cingulate pioneering axons are projecting to homotopic targets in the contralateral hemisphere. Midline crossing of callosal axons continues during early postnatal stages in mice.

Another important aspect of commissure development that could also account for evolutionary events that led to commissure diversification involves axon guidance and targeting. Following the induction and patterning of the telencephalic midline, growing commissural axons are channeled toward and across the midline by a number of glial cell populations present throughout mammal species (Silver et al., 1982; Cummings et al., 1997; Pires-Neto et al., 1998; Lent et al., 2005). For example, the indusium griseum glia (IGG) and the glial wedge form dorsomedial and ventrolateral boundaries for growing callosal axons, respectively, while the midline zipper glia (MZG) demarcate a ventromedial boundary (Figure 5; Silver et al., 1993; Shu and Richards, 2001; Shu et al., 2003). In mice, glial wedge cells are born around embryonic day (E) 13 and, while retaining their cell bodies in the medial aspect of the lateral ventricle, they extend processes that cluster into a wedge shape that coincides with the boundary between pallial and subpallial domains (cortico-septal boundary, Figure 5A). This cell population, together with the IGG, guide growing axons by expressing chemorepellent molecules such as Slit2, Wnt5a, and Draxin, thus preventing callosal axons from coursing ventrally into septal territory (Shu and Richards, 2001; Keeble et al., 2006; Islam et al., 2009; Unni et al., 2012). By E15, pioneer axons from the cingulate cortex first cross the midline (Koester and O'Leary, 1994; Rash and Richards, 2001), followed by isocortical axons, which fasciculate with them to cross the midline approximately 1 day later (Figures 5A,B). Another cell population that participates in the guidance of callosal axons at the midline is the subcallosal sling (Figure 5C), also referred to as the callosal corridor, a transient neuronal population that lies at the ventral border of the corpus callosum (Silver et al., 1982, 1993; Silver and Ogawa, 1983; Hankin et al., 1988; Shu et al., 2003; Niquille et al., 2009; Benadiba et al., 2012). These cells express Sema3c, which acts as an attractant of pioneer axons from the cingulate cortex through interaction with its receptor Nrp1 (Niquille et al., 2009; Piper et al., 2009).

After crossing the midline, callosal axons grow into the contralateral hemisphere and innervate homotopic (Yorke and Caviness, 1975; Krubitzer et al., 1998; Rash and Richards, 2001; Hofer and Frahm, 2006), and heterotopic regions of the cortex (Boyd et al., 1971; Kretz and Rager, 1990; Aboitiz and Montiel, 2003). Histological studies in mice have revealed a dorso-ventral segregation of callosal axons according to the medio-lateral position of their cell-bodies within the cortex (Richards et al., 2004; Zhou et al., 2013). A similar situation has been described in humans using magnetic resonance imaging, where callosal fibers originating at different medio-lateral positions retain a dorso-ventral parcellation within the rostro-caudal axis (Abe et al., 2004; Tovar-Moll et al., 2007; Chao et al., 2009; Fabri et al., 2011; Fabri and Polonara, 2013). Thus, a highly refined topographic organization of axons at the midline is a shared feature of commissural systems. The primary somatosensory and visual cortices of rodents send callosal projections to homotopic and heterotopic regions, with a distinct axonal arborization at the border between primary and secondary corresponding areas in the contralateral hemisphere (Wise and Jones, 1976; Ivy and Killackey, 1981; Koralek and Killackey, 1990; Mizuno et al., 2007; Wang et al., 2007). Formation of these contralateral projections occurs mostly during postnatal stages (Wise and Jones, 1976; Wang et al., 2007; Mizuno et al., 2010), and depends on sensory-evoked and spontaneous neural activity during a critical period. Early deprivation of the sensory periphery or thalamic lesions during the first postnatal week in rodents prevents normal development of callosal projections (Innocenti and Frost, 1979; Olavarria et al., 1987; Koralek and Killackey, 1990; Innocenti and Price, 2005). Similarly, disruption of electrical activity directly in callosal neurons results in disrupted contralateral projections (Mizuno et al., 2007, 2010; Wang et al., 2007), suggesting that early experience plays an instructive role in the precise targeting of contralateral axons (Huang et al., 2013; Suárez et al., 2014). Thus, additional developmental processes that may have influenced the origin and diversification of mammalian commissures include precise temporal and spatial interactions between glial cells and neurons, production of axon guidance ligands and expression of receptors, and early spontaneous and sensory-evoked neuronal activity.

## Conclusion

In the context of evolution and development of forebrain commissures, a number of brain features can be distinguished as highly conserved throughout vertebrates, the first being a requirement for interhemispheric communication of the two halves of the CNS. The presence of commissural systems throughout bilaterians reflects a computational requirement of interhemispheric coordination for normal behavior. Second, the conservation in vertebrates of a defined set morphogen expression at the telencephalic midline indicates an important developmental event that directs both the identity patterning of brain areas and wiring of commissural axons. Third, another feature of commissural systems shared by vertebrates is the co-occurrence of decussating fibers that project to heterotopic regions with commissural fibers connecting homotopic regions between hemispheres. Moreover, the presence of profuse heterotopic projections in forebrain commissural pathways of early-branched vertebrates suggests that homotopic projections arose as a refinement of the former kind. Finally, a topographical arrangement of axons within the commissural tracts according the place of origin of their cell bodies can also be recognized as a general feature of commissural systems. Moreover, the origin of new commissures, such as the pallial commissure in early tetrapods and the corpus callosum in eutherian mammals, seems to involve the rerouting of a specific population of topographically arranged axons through preexistent commissural substrates. Such examples of axonal rearrangement can be found in congenital cases of callosal malformations in humans (Tovar-Moll et al., 2007, 2014; Wahl et al., 2009).

Although there is currently little evidence to allow speculation about the precise mechanisms that led to the evolution of the corpus callosum in eutherian mammals, an evolutionary developmental approach integrating current gene manipulation techniques in carefully selected animal models may shed light on this fascinating topic.

### Conflict of interest statement

The authors declare that the research was conducted in the absence of any commercial or financial relationships that could be construed as a potential conflict of interest.
